# Use of Psychoeducation for Psychotic Disorder Patients Treated With Modern, Long-Acting, Injected Antipsychotics

**DOI:** 10.3389/fpsyt.2021.804612

**Published:** 2021-12-15

**Authors:** Antonio Ventriglio, Annamaria Petito, João Maurício Castaldelli-Maia, Julio Torales, Valeria Sannicandro, Eleonora Milano, Salvatore Iuso, Antonello Bellomo

**Affiliations:** ^1^Department of Clinical and Experimental Medicine, University of Foggia, Foggia, Italy; ^2^Department of Neuroscience, Medical School, Fundação do ABC, Santo André, Brazil; ^3^Department of Psychiatry, School of Medical Sciences, National University of Asunción, Asunción, Paraguay

**Keywords:** long-acting antipsychotics, psychotic disorders, metabolic changes, psychoeducation, treatment-adherence

## Abstract

**Introduction:** There is an increased risk of adverse metabolic effects of some modern antipsychotic drugs, and concern that long-acting, injected preparations of them may increase such risk. We now report on clinical and metabolic outcomes in patient-subjects diagnosed with affective and non-affective psychotic disorders following exposure to psychoeducation on metabolic risks of modern antipsychotics followed by treatment with long-acting atypical injected antipsychotics over 6-months.

**Materials and Methods:** 85 psychotic disorder outpatients (42 affective [AP]; 43 non-affective [NAP]) at the University of Foggia were treated with long-acting, injected, second-generation antipsychotics in association with a set of psychoeducational sessions concerning general health and potential effects of antipsychotic drug treatments. They were evaluated at baseline and six months.

**Results:** Initially, NAP subjects reported higher ratings of positive and negative symptoms than AP subjects, were more likely to receive risperidone or paliperidone, with higher CPZ-eq doses of antipsychotics (294.0 ± 77.8 vs. 229.3 ± 95.8 mg/day), and shorter QTc electrocardiographic recovery intervals. During the 6-month follow-up, ratings of treatment-adherence improved through overall (+8.75%), and symptom-ratings decreased (−7.57%) as did Body-Mass Index (−2.40%; all *p* ≤ 0.001). Moreover, serum levels of fasting glucose, hemoglobin glycosylation, cholesterol and prolactin concentrations all decreased, with little difference between subjects with AP vs. NAP.

**Discussion and Conclusions:** A psychoeducational program was associated with consistent improvement in psychotic symptoms and several metabolic and physiological measures, as well as with treatment-adherence during six months of treatment with long-acting, injected, second-generation antipsychotics, in association with both affective and non-affective psychotic disorders.

## Introduction

Major mental disorders are associated with increased risk of morbidity and mortality due to the illnesses themselves and their treatments (1). The prevalence of metabolic syndrome (MS) among schizophrenia patients may range 37-63% with a relative risk (RR) of 2–3 in patients compared to general population, as well as MS in bipolar disorder patients ranges 30–49% with a RR of 1.5-2 (2). Consequently, it has been largely described an increased risk of death from medical causes in schizophrenia and 20% (10–15 years) shorter lifespan (2, 3); similarly, affective psychoses are associated with higher standardized mortality ratios from medical causes than general population ranging 1.9-2.1 (2, 3). Many factors including poor life-style and food intake, poor attention to health needs and side effects of psychotropic drugs may impact on the metabolic outcome of patients affected by psychoses (3). Currently, the employment of second- and third- generation antipsychotics, such as serotonin- dopamine antagonists or dopamine partial agonists, are clinically preferred since are more effective on negative, affective and cognitive symptoms of psychosis (4). Nonetheless, newer antipsychotics may induce increases in body-weight, insulin- resistance with long- term adverse physiological effects including type−2- diabetes mellitus, hypertension and dyslipidemia, in addition to obesity (4–6). Also, it has been largely described that the prevalence of metabolic syndrome in patients treated with antipsychotics is approximately 40% (33.8–42.1%) in contrast to rates of 10.4–12.2% among psychosis un-medicated patients: some atypical antipsychotics such as olanzapine and risperidone were associated to higher weight gain among non-clozapine second generation antipsychotics, as well as risperidone and amisulpride were responsible for increasing serum prolactin (7).

These metabolic issues are potentially life-threatening effects and need to be carefully assessed and treated by clinicians (8, 9). Also, strategies of prevention need to be employed and awareness regarding their own general health needs to be addressed among these patients (10). International organizations have proposed guidelines for screening and preventing metabolic issues in patients treated with antipsychotics suggesting physical monitoring and psycho-education in the long-term treatment (11, 12).

Physical activity may be a relevant therapeutic intervention for people with severe mental disorders and life style-related medical issues: this has been confirmed among schizophrenia-spectrum disorders patients as well as those affected by major depressive disorder and bipolar disorder (2). In addition, life-style factors such as tobacco smoking, unhealthy dietary patterns, poor sleep, together with poor physical activity have been found to be associated to higher risk of mental illness and poorer outcome of illness: these data suggest that life-style factors need to be addressed within mental health care (12).

We describe the impact of a repeated, systematic, prospective psycho- educational intervention on clinical and metabolic outcomes of patients affected by stable affective (APs) and non-affective psychoses (NAPs) treated with long-acting atypical antipsychotics over 6-months. We compared psychopathology and medical parameters of interest between the diagnostic groups at baseline and at the end of the program aimed at increasing awareness of the general health, improving diet and exercise, and limiting obesity and other side effects.

## Materials and Methods

### Subjects

85 outpatients affected by Affective Psychoses (*n* = 42; APs: Schizoaffective Disorders, Not Otherwise Specified Psychoses) and Non-Affective Psychoses (*n* = 43; NAPs: Schizophrenia, Delusional Disorders) attending the Unit of Psychiatric at University of Foggia and treated with long-acting atypical antipsychotics, at stable doses, have been recruited and assessed at intake (T1) and 6 months (T2) for psychopathology, treatments, adherence, and monitored for anthropometric and electrocardiographic measures. Also, they received a systematized, repeated psycho-education about physical health. Diagnoses met DSM-5 (The Diagnostic and Statistical Manual of Mental Disorders, Fifth Edition), confirmed with the Mini-International Neuropsychiatric Interview (MINI) performed by consensus of experienced clinicians (AV, AP, and SI) (13, 14). This is a real-world study based on a straightforward clinical assessment with easy and reliable tools.

Patients were voluntarily recruited, clinically treated and followed at the Psychiatric Outpatient Services of University of Foggia Medical Center in 2014–2018, as part of an approved PhD Program in Clinical and Experimental Medicine at University of Foggia. All participants provided written, informed consent and the intervention was approved by the Medical Center Ethical Review Committee. Acutely psychiatrically or medically ill patients were excluded and we recruited those reporting a stable phase of illness in the previous three months and treated with a stable long-acting antipsychotic treatment clinically determined and followed for six months.

### Assessment and Monitoring Procedures

Patients were assessed and monitored at intake (T1) and 6 months (T2) of follow-up, employing standardized methods. Clinical assessment, treatment selection and physical monitoring have been run by psychiatrists (AV, AB, VS, EM), whereas psychoeducation and rating scales have been delivered by two expert psychologists (AP, SI). Required information was gathered at intake and repeatedly during the follow-up. Electrocardiograms (with the record of QTc interval) were performed at intake and final visit as well as anthropometrics (height, weight, waist circumference, body mass index [BMI]), vital signs (blood pressure and pulse rate) and serum assays (serum lipids, carbohydrates, prolactin) which were carried out consistently by the University Medical Center clinical laboratory.

Antipsychotic drug- doses were converted to mg/day chlorpromazine-equivalents according to suggested conversion formulas (15, 16). Adherence to treatment was rated with the 30-item Drug Attitude Inventory (DAI-30) at intake and six months (17). Psychopathology including positive, negative and general symptoms were rated with the Positive and Negative Syndrome Scale (PANSS) (18) and the Brief Psychiatric Rating Scale (BPRS) (19) at intake and six months; each investigator has been trained in order to employ the tools correctly and levels of inter-rater agreement were calculated (κ-statistic ≥0.94).

### Psychoeducational Program

The repeated, systematic, prospective psycho- educational intervention was provided to all subjects once/month in conjunction with the administration of monthly long-acting injectable treatment. It included six sessions concerning psychiatric and general health, diet, exercise, weight-control, current treatment, following methods recommended by Littrell and colleagues (20). Patients were also trained to regularly measure vital signs (blood pressure, pulse rate) anthropometric parameters (weight, body-mass index [BMI], waist circumference) and advised about food-selection, healthy diets, daily physical exercise. Motivational modules regarding daily activity were performed.

In this trial no control condition was included.

### Data Analysis

Data analyses were performed with standard, commercial, statistical software (Statview® SAS Institute, Cary, NC; Stata®, StataCorp, College Station, TX). Data were presented as means or percentages (including %-changes) with 95%- Confidence Intervals- or standard deviations (as %-changes), compared between diagnosis by ***t***-test or χ^2^. We also carried out repeated-measures ANOVA to evaluate changes in parameters of interest over time. Findings are considered statistically significant with two-tailed *p* ≤ 0.05.

## Results

### Sample Characteristics at Intake

85 adult outpatients affected by Affective Psychoses (*n* = 42; APs: Schizoaffective Disorders, Not Otherwise Specified Psychoses) and Non-Affective Psychoses (*n* = 43; NAPs: Schizophrenia, Delusional Disorders) were recruited in the 2014–2018 period, treated and followed for six months. Those with NAPs or APs included 30.2 and 52.3% women respectively (*p* = 0.048), with overall age-at-intake of 43.8 ± 12.2 vs. 40.3 ± 11.6 years. Demographic characteristics were similar through the diagnostic subgroups (Table 1). Psychopathological symptoms rated with PANSS and BPRS scales were slightly higher among NAPs vs. APs patients (63.2 ± 30.3 vs. 50.4 ± 18.2 [PANSS; *p* = 0.022] and 42.3 ± 14.4 vs. 36.9 ± 12.3 [BPRS; *p* = 0.068]).

**Table 1:** Subject characteristics at intake.

**Measures**	**Means [95%CI]**			***p*-value**
	**Non-affective psychoses**	**Affective psychoses**	**All pychoses**	
Number (n)	43	42	85	-
Female (%)	30.2 [17.2–46.1]	52.4 [36.4–68.0]	41.2 [30.6–52.4]	**0.048**
Age (years)	43.8 [39.6–48.0]	40.3 [36.7–43.9]	42.0 [39.3–41.7]	0.212
Married (%)	9.30 [2.59–22.1]	21.4 [10.3–36.8]	15.3 [8.40–24.7]	0.142
Employed (%)	6.98 [1.46–19.9]	19.1 [8.60–34.1]	12.9 [6.64–22.0]	0.117
*Initial morbidity ratings*				
PANSS (total)	63.2 [53.9–72.5]	50.4 [44.7–56.1]	56.9 [51.4–62.4]	**0.022**
BPRS (total)	42.3 [37.9–46.7]	36.9 [33.1–40.7]	39.6 36.7–42.5[]	0.068
*Antipsychotic use* (%)				
Risperidone-LAI	27.9 [15.3–43.7]	9.52 [2.66–22.6]	18.8 [11.2–28.8]	**0.030**
Paliperidone-LAI	72.1 [56.3–84.7]	42.9 [27.7–59.0]	57.6 [46.4–68.3]	**<0.001**
Olanzapine-LAI	0.00 [0.00–0.00]	16.7 [6.97–31.4]	8.24 [3.38–16.2]	**0.005**
Aripiprazole-LAI	0.00 [0.00–0.00	31.0 [17.6–31.4]	15.3 [8.40–24.7]	**<0.001**
Mean dose (CPZ-eq mg/day)	294 [270–318]	229 [199–259]	262 [242–282]	**0.001**
DAI-30 score	10.9 [10.2–11.6]	11.1 [10.4–11.8]	11.0 [10.5–11.5]	0.765
Body-mass index (BMI, kg/m^2^)	28.4 [26.7–30.1]	28.1 [26.0–30.2]	28.2 [26.9–29.5]	0.792
Waist circumference (cm)	103 [98.3–108]	101 [95.7–106]	102 [98.5–105]	0.668
*Blood pressure & pulse*				
Systolic (mm Hg)	119 [116–122]	122 [118–126]	118 [116–120]	0.262
Diastolic (mm Hg)	76.8 [74.2–79.4]	75.3 [72.4–78.2]	76.3 [74.4–78.2]	0.191
Pulse rate (per min)	85.7 [82.6–88.8]	83.4 [79.6–85.6]	83.4 [81.5–85.3]	0.399
QTc interval (msec)	407 [398–416]	420 [413–427]	413 [407–419]	**0.020**
*Carbohydrates* (serum)				
Fasting blood glucose	94.7 [90.1–99.3]	94.3 [89.4–99.2]	94.5 [91.2–97.8]	0.897
Hemoglobin glycosylation (%)	6.28 [6.02–6.55]	6.15 [5.96–6.34]	6.09 [6.03–6.15]	0.442
*Serum lipids* (mg/dL)				
Triglycerides	143 [124–162]	150 [120–180]	146 [129–163]	0.710
Total cholesterol	188 [178–198]	196 [184–208]	192 [184–200]	0.335
Low-density cholesterol (LDL)	125 [117–133]	123 [110–136]	124 [117–131]	0.859
High-density cholesterol (HDL)	45.9 [42.5–49.3]	49.8 [45.7–54.3]	47.8 [45.1–50.5]	0.150
Serum prolactin (μg/L)	45.1 [34.6–55.6]	40.4 [27.9–52.9]	42.8 [34.8–50.8]	0.563

***Boldface**: Factors significantly different at intake by diagnostic type, by t-test or χ^2^ (p <0.05)*.

*PANSS, Positive and Negative Syndrome Scale; BPRS, Brief Psychiatric Rating Scale; CPZ-eq, chlorpromazine-equivalent mg/day; DAI-30, 30-item Drug Attitude Inventory*.

Long-acting treatments, clinically selected, were exclusively based on SDA agents (injectable- risperidone [27.9%] and - paliperidone [72.0%]) for NAP patients and also included olanzapine and aripiprazole for AP patients ranking: paliperidone (42.8%)> aripiprazole (30.9%) > olanzapine (16.6%)> risperidone (9.52%) (*p* = 0.000). Average daily doses of antipsychotic (all converted to chlorpromazine- equivalent milligrams for comparison) were much higher among NAP vs. AP patients (294.0 ± 77.8 vs. 229.3 ± 95.8; *p* = 0.001).

Baseline anthropometrics, serum parameters and vital signs were somewhat similar between diagnostic groups and recorded QTc electrocardiographic interval was slightly higher among AP vs. NAP patients (419.9 ± 22.3 vs. 406.6 ± 29.2; *p* = 0.020) (Table 1).

### Follow-Up Assessment

Measures changes from baseline (T1) were recorded after 6 months (T2) of follow-up and psycho- educational program. Changes were computed as means ± standard deviations (not shown) but also presented in a more informative manner as %-changes with standard deviations: [(parameter at T1- parameter at T2)/ parameter at T1]^*^100. Findings for all cases (*N* = 85) at 6-months showed a significant improvement of adherence-rate (DAI-30; +8.75%), reduction of psychopathological ratings (−7.57 % at PANSS and −6.45 % at BPRS), decrease of BMI (-2.40%) as well as QTc Interval (-0.20%), fasting glucose (−2.54%), hemoglobin glycosylation (−3.47%), total cholesterol with an increase of high density lipoproteins (−3.98 and +34.8 % respectively), reduction in serum prolactin (-4.81%) (0.000 ≤ *p* ≤ 0.002). Measures changes have shown little differences among diagnoses. PANSS score decreased highly among NAP vs. AP patients (−9.54 vs. −3.76%) as well as hemoglobin glycosylation (−3.84 vs. −2.76%), QTc interval (−0.209 vs. −0.203%) and serum prolactin (−4.97 vs. −4.49%) (0.006 ≤ *p* ≤ 0.035) (Table 2). Finally, we computed changes in measures among the different long-acting treatments over time: all measures did not differ among the treatments (0.068 ≤ *p* ≤ 0.981; not shown).

**Table 2:** Changes of measures over six months of long-acting antipsychotic treatment and psychoeducation.

**Measures**	**Changes (%) with SD**					
	**All cases**		***p*-values**		**Non-affective psychoses**	**Affective psychoses**	***p*-values**
PANSS score	−7.57	9.44	**<0.001**		−9.54 [9.03]	−3.76 [9.19]	**0.006**
BPRS score	−6.45	9.68	**<0.001**		−6.36 [11.3]	−6.63 [5.09]	0.079
DAI-30 score	+8.75	5.93	**<0.001**		+9.36 [5.86]	+7.58 [5.98]	0.194
BMI (kg/m^2^)	−2.40	4.77	**<0.001**		−2.33 [5.31]	−2.77 [3.58]	0.227
Waist (cm)	−58.0	36.6	0.646		−59.0 [34.0]	−56.2 [41.6]	0.185
QTc	−0.20	0.01	**0.002**		−0.20 [0.01]	−0.20 [0.01]	**0.039**
Diastolic BP	+0.71	14.6	0.841		+0.71 [13.6]	+0.70 [16.8]	0.064
Systolic BP	+2.71	12.2	0.135		+2.04 [11.2]	+4.00 [14.2]	0.661
Pulse rate	+2.05	16.3	0.605		+2.35 [15.7]	+1.49 [17.8]	0.594
Fasting glucose	−2.54	8.30	**0.001**		−3.15 [7.36]	−1.36 [9.89]	0.953
Hemoglobin glycosylation	−3.47	3.87	**<0.001**		−3.84 [3.59]	−2.76 [4.34]	**0.007**
Total cholesterol	−3.98	9.54	**<0.001**		−3.33 [9.87]	−5.24 [8.92]	0.557
LDL cholesterol	−0.87	0.27	0.265		−0.86 [0.25]	−0.89 [0.30]	0.858
HDL cholesterol	+34.8	55.1	**<0.001**		+27.2 [46.7]	+49.3 [67.0]	0.186
Triglycerides	−12.41	44.0	0.166		−11.8 [44.6]	−13.4 [43.6]	0.871
Serum prolactin	−4.81	11.0	**0.000**		−4.97 [13.3]	−4.49 [4.43]	**0.035**

*The 85 APs and NAPs patient-subjects were assessed clinically at baseline and 6 months, changes in measures were tested by repeated measures ANOVA*.

*SD, standard deviation; PANSS, Positive and Negative Syndrome Scale; BPRS, Brief Psychiatric Rating Scale; CPZ-eq, chlorpromazine-equivalent mg/day; DAI-30, 30-item Drug Attitude Inventory; BMI, Body-Mass Index; HDL, High-Density Lipids (cholesterol). LDL, Low-Density Lipids*.

***Boldface**: Factors significantly different*.

## Discussion and Conclusion

This study aimed to test the impact of a psychoeducational program on general health among 85 patient-subjects diagnosed with clinically stable affective and non-affective psychoses treated with long-acting, injected, second-generation drugs, as determined by treating physicians. Changes in clinical, anthropometric and physiological measures over six months of treatment exposure were measured.

Clinicians preferred risperidone and paliperidone antipsychotics for the long-term treatment of patient-subjects diagnosed with schizophrenia or other non-affective psychoses, possibly due to their relatively high affinity at dopamine D_2_-receptors and their reported benefits on both positive and negative psychotic symptoms (21). Olanzapine and aripiprazole were preferred with affective psychoses since they may have mood-stabilizing effects (22). Subjects with non-affective psychoses were also given relatively high CPZ-eq doses of antipsychotics. Among few initial differences between subjects with affective and non-affective disorders, the QTc electrocardiographic interval was slightly higher with affective disorders (Table 1). It is of interest that patients recruited in the study were not affected by serious cardiological conditions and a small number of subjects were taking anti-hypertensive drugs (27.2%; not described for heterogeneity of data). Variations of QTc intervals among patients may also reflect an additional individual variability (23).

By six-months of treatment that involved close clinical follow-up and continued psychoeducational intervention was associated with several moderate, but favorable changes (Table 2). These include improved adherence ratings, even above those expected with injected, long-acting drugs: this reflects the evidence that joining a psycho-educational program may increase the personal awareness about illness and improve patients' attitudes regarding its treatment (24). Also, improvements in psychotic-symptoms ratings (PANSS and BPRS) may reflect benefits of the medication provided and perhaps added benefits associated with the psychoeducational intervention aimed to increase patients' *insight* (25). Improvements in BMI, carbohydrates, hemoglobin glycosylation, serum lipids and prolactin concentration may in part reflect changes in life-style, diet and physical exercise, all as encouraged by the psychoeducation intervention (26). In addition, participants' attitudes toward medications and their adherence to scheduled injections improved as reflected in ratings with the DAI-30.

### Limitations

Cause-and-effect relationships involved in the observed changes are not clear without a control condition lacking psychoeducation. In fact, the comparison with a control-group of patients treated with long-acting medications with no psychoeducation (as well as patients treated with oral antipsychotics and psychoeducation) would add more evidence on the cause-and-effect relationships between their clinical outcomes and psychoeducational intervention. Also, it is notable that differences in baseline measures and their changes with treatment related to the type of psychotic illness were somewhat negligible (Tables 1, 2). This study is limited by the relatively small number of subjects, only 6 months of treatment and follow-up, the lack of a comparison condition without a psychoeducational component, and lack of blinding to diagnosis and treatment. Nonetheless, similar improvements in clinical and metabolic status of severe mentally ill patients given the same psycho-educational program have been confirmed in our previous study conducted in 2014 (3).

In conclusion, the findings suggest favorable changes in clinical and metabolic status among severely ill psychotic outpatients treated with modern, long-acting, injected antipsychotic drugs combined with close clinical follow-up and ongoing psychoeducation. We suggest that such interventions may contribute to improving clinical and medical outcomes in psychotic disorders and limit mortality-risk, and conclude that they require further, controlled testing.

## Data Availability Statement

The raw data supporting the conclusions of this article will be made available by the authors, without undue reservation.

## Ethics Statement

This study involving human participants has been reviewed and approved by University of Foggia. The patients/participants provided their written informed consent to participate in this study.

## Author Contributions

AV and AB: each authors has contributed to the development of the report. AV, AP, VS, EM, and SI: reviewing clinical and pharmacy records. AV, JC-M, and JT: analyzing data. All authors contributed to the article and approved the submitted version.

## Conflict of Interest

The authors declare that the research was conducted in the absence of any commercial or financial relationships that could be construed as a potential conflict of interest.

## Publisher's Note

All claims expressed in this article are solely those of the authors and do not necessarily represent those of their affiliated organizations, or those of the publisher, the editors and the reviewers. Any product that may be evaluated in this article, or claim that may be made by its manufacturer, is not guaranteed or endorsed by the publisher.
